# Blood regulator of G protein signalling 1 as a potential prognostic biomarker in surgical nonsmall cell lung cancer patients: Correlation with clinical features and survival

**DOI:** 10.1111/crj.13712

**Published:** 2023-12-11

**Authors:** Liping Wang, Hui Zhang, Xinliang Gu, Ying Wang

**Affiliations:** ^1^ Department of Oncology Baotou Cancer Hospital Baotou China; ^2^ Department of Medical Iconography Baotou Cancer Hospital Baotou China

**Keywords:** clinical characteristics, nonsmall cell lung cancer, prognostic value, regulator of G protein signalling 1, tumour‐infiltrating immune cells

## Abstract

**Introduction:**

Regulator of G protein signalling 1 (RGS1) closely regulates malignant phenotypes and tumour immunity in several cancers, while its clinical value in nonsmall cell lung cancer (NSCLC) is by far rarely reported. Consequently, this study aimed to explore the linkage of blood RGS1 with clinical features and prognosis in surgical NSCLC patients.

**Methods:**

Two‐hundred and ten surgical NSCLC patients were consecutively enrolled in this study, whose RGS1 in peripheral blood mononuclear cells was determined before treatment via reverse transcriptional‐quantitative polymerase chain reaction. Additionally, the blood RGS1 was also collected from 30 healthy controls (HCs).

**Results:**

Blood RGS1 was increased in NSCLC patients compared with HCs (*P* < 0.001). Elevated blood RGS1 was related to lymph node (LYN) metastasis (*P* = 0.001), higher tumour‐nodes‐metastasis (TNM) stage (*P* = 0.004), neoadjuvant chemotherapy administration (*P* = 0.044), shortened accumulative disease‐free survival (DFS) (*P* = 0.008) and overall survival (OS) (*P* = 0.013) in NSCLC patients. A multivariate Cox's regression analysis showed that blood RGS1 high expression could independently reflect shortened DFS (hazard ratio = 1.499, *P* = 0.023), whereas it could not independently predict OS (*P* > 0.050). Furthermore, blood RGS1 high expression was associated with shortened OS (*P* = 0.020) in patients with neoadjuvant therapy and with worse DFS (*P* = 0.028) and OS (*P* = 0.026) in patients with adjuvant therapy, while blood RGS1 was not linked with DFS or OS in patients without neoadjuvant or adjuvant therapy (all *P* > 0.050).

**Conclusion:**

Elevated blood RGS1 correlates with LYN metastasis, neoadjuvant chemotherapy administration, worse DFS and OS, which might serve as a useful prognostic biomarker for surgical NSCLC patients.

## Graphical abstract

This study determined regulator of G protein signalling 1 (RGS1) in peripheral blood mononuclear cells of 210 surgical nonsmall cell lung cancer (NSCLC) patients and 30 healthy controls (HCs). Interestingly, blood RGS1 was increased in NSCLC patients versus HCs (*P* < 0.001). Elevated blood RGS1 was related to lymph node metastasis (*P* = 0.001), higher tumour‐nodes‐metastasis stage (*P* = 0.004), neoadjuvant chemotherapy administration (*P* = 0.044), shortened disease‐free survival (DFS) (*P* = 0.008) and overall survival (OS) (*P* = 0.013) in NSCLC patients. A multivariate Cox's regression analysis showed that elevated blood RGS1 could independently reflect shortened DFS (hazard ratio = 1.499, *P* = 0.023). Furthermore, increased blood RGS1 was associated with shortened OS (*P* = 0.020) in patients with neoadjuvant therapy and with worse DFS (*P* = 0.028) and OS (*P* = 0.026) in patients with adjuvant therapy, while blood RGS1 was not linked with DFS or OS in patients without neoadjuvant or adjuvant therapy (all *P* > 0.050). In summary, blood RGS1 might serve as a useful prognostic biomarker for surgical NSCLC patients.

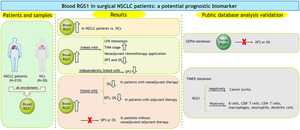

## 1 INTRODUCTION

Nonsmall cell lung cancer (NSCLC), accounting for nearly 85% of lung cancer cases, is by far the second most frequently diagnosed malignancy and the main cause of cancer‐related death worldwide due to its high‐progressive and late‐diagnosis characteristics.^1^, ^2^, ^3^ In recent years, novel treatment patterns (including targeted treatment, immunotherapy, etc.) have been developed to improve the survival of NSCLC patients.^4^, ^5^ Regrettably, the prognosis of NSCLC patients is quite heterogeneous, whose 5‐year overall survival (OS) rate ranges from 68%–92% in tumour‐nodes‐metastasis (TNM) stage I patients to nearly 0%–10% in TNM stage IV patients.^6^, ^7^ Consequently, identifying potential biomarkers with the clinical utility to recognise different prognoses in NSCLC patients conduces to optimise treatment therapies and improve their survival outcomes.

Regulator of G protein signalling 1 (RGS1), belonging to the R4 subfamily of RGS proteins, is an inhibitor of G protein‐coupled receptors (GPCRs) that closely involves in malignant phenotypes of several cancers, such as osteosarcoma and cervical cancer.^8^, ^9^, ^10^ For instance, one previous study discloses that RGS1 facilitates cell proliferation, migration and invasion but retards cell apoptosis of osteosarcoma cells.^10^ From the clinical aspect, another study indicates that the aberrant expression of tumour RGS1 in several cancers (including liver hepatocellular carcinoma, pancreatic cancer and thymoma) is related to poor prognosis.^11^ Moreover, plentiful evidences reveal that RGS1 exerts an essential role in regulating tumour immunity, which further promotes tumourigenesis, tumour progression and drug resistance.^12^, ^13^, ^14^ For example, one study shows that RGS1 knockdown accelerates the infiltration and survival of cytotoxic T lymphocytes; meanwhile, elevated RGS1 reduces the trafficking of tumour‐specific T‐cells to tumours, which is correlated with shortened survival of breast and lung cancer patients.^12^ Based on the aforementioned evidences, RGS1 exerts the carcinogenic effect through modulating malignant phenotypes and the chemotaxis of immune cells; subsequently, it is speculated that blood RGS1 might possess a prognostic value in NSCLC patients as well.

Hence, this study determined blood RGS1 expression in 210 surgical NSCLC patients and 30 healthy controls (HCs), intending to explore the association of blood RGS1 with clinical characteristics and its prognostic value in NSCLC patients.

## 2 MATERIALS AND METHODS

### 2.1 Participants

Two‐hundred and ten primary NSCLC patients receiving surgical resection in our hospital between July 2016 and July 2021 were consecutively enrolled in the current study. Patients with the following criteria were eligible for enrollment: 1. pathologically diagnosed as primary NSCLC, 2. aged above 18 years, 3. planned for surgical resection of tumour and 4. willing to provide the specimen and data for analysis. Patients who met one of the following criteria were excluded from this study: 1. with recurrent NSCLC, 2. complicated with other primary malignancies and 3. with distant metastases. Simultaneously, this study also enrolled 30 age‐ and gender‐matched HCs. All participants signed informed consent. This study was reviewed by the Ethics Community of Baotou Cancer Hospital.

### 2.2 Data record and sample collection

The data in NSCLC patients were recorded in detail, which included demographics, medical histories, disease information and treatments. The peripheral blood mononuclear cells (PBMCs) of NSCLC patients before neoadjuvant treatment (if they had) or surgery were collected. Meanwhile, the PBMCs of HCs were also collected after enrollment.

### 2.3 Blood RGS1 detection

RGS1 in the PBMCs was analysed by reverse transcriptional‐quantitative polymerase chain reaction (RT‐qPCR). PBMC RNA extraction was conducted by TRIzol™ Reagent (Thermo Fisher Scientific), then reversely transcribed into complement DNA with PrimeScript™ RT Master Mix (Perfect Real Time) (Takara, China). Next, quantitative PCR was carried out with TB Green™ Fast qPCR Mix (Takara, China). Each experiment was conducted according to the kits' instructions. Blood RGS1 expression was assessed by the 2^‐ΔΔct^ formula^15^ with glyceraldehyde 3‐phosphate dehydrogenase as the internal control. The primers were in accordance to previous studies.^16^, ^17^

### 2.4 Treatment

All patients received proper treatment, which was based on patients' disease condition and willing, as well as clinicians' advice. The treatment information of NSCLC patients (including neoadjuvant and adjuvant treatment, if they underwent them) was recorded in detail. The neoadjuvant or adjuvant treatment regimens were cisplatin combined with navelbine (NP), gemcitabine (GP), docetaxel (DP) or paclitaxel (TP).

### 2.5 Follow‐up

All NSCLC patients underwent regular follow‐up until 31 May 2022. The median and range of follow‐up was 39.7 (7.3–65.8) months. Based on the follow‐up data, the disease‐free survival (DFS) and OS were calculated.

### 2.6 Statistical analysis

Statistical analysis and figure plotting were conducted by SPSS V.24.0 and GraphPad Prism V.8.01, respectively. Comparison of blood RGS1 between two groups was conducted by Mann–Whitney *U* test. Comparison of blood RGS1 among three or more groups was performed with Kruskal–Wallis test. Association analysis was completed by Spearman's rank correlation. DFS and OS were shown with Kaplan–Meier curves. DFS or OS between two groups was compared with log‐rank test. Distinguish abilities of RGS1 were assessed by receiver‐operating characteristic (ROC) curves and area under the curve (AUC). Factors of DFS and OS were estimated with univariate and forward stepwise multivariate Cox's regression analysis. Statistical significance was considered if *P*‐value was less than 0.05.

## 3 RESULTS

### 3.1 Characteristics of NSCLC patients

All 210 NSCLC patients underwent surgical resection in the present study, consisting of 165 (78.6%) males and 45 (21.4%) females, with a mean age of 61.4 ± 11.2 years (Table 1). A respective of 150 (71.4%) patients and 60 (28.6%) patients were evaluated with Eastern Cooperative Oncology Group Performance Status (ECOG PS) score 0 and 1. Concerning the TNM stage, 36 (17.1%), 87 (41.4%) and 87 (41.4%) patients were assessed as TNM stage I, II and III, correspondingly. Furthermore, 81 (38.6%) and 162 (77.1%) patients received neoadjuvant chemotherapy and adjuvant chemotherapy, accordingly. The specific clinical characteristics of NSCLC patients were exhibited in Table 1.

**TABLE 1:** Characteristics of NSCLC patients.

Items	NSCLC patients (*N =* 210)
Age (years), mean ± SD	61.4 ± 11.2
Gender, *n* (%)	
Male	165 (78.6)
Female	45 (21.4)
Smoke, *n* (%)	82 (39.0)
Drink, *n* (%)	73 (34.8)
Hypertension, *n* (%)	62 (29.5)
Hyperlipidemia, *n* (%)	68 (32.4)
Diabetes, *n* (%)	37 (17.6)
Subtype, *n* (%)	
Adenocarcinoma	102 (48.6)
Squamous cell carcinoma	97 (46.2)
Adenosquamous carcinoma	11 (5.2)
ECOG PS score, *n* (%)	
0	150 (71.4)
1	60 (28.6)
Tumour differentiation, *n* (%)	
Well	33 (15.7)
Moderate	110 (52.4)
Poor	67 (31.9)
Tumour size, *n* (%)	
≤ 5 cm	99 (47.1)
> 5 cm	111 (52.9)
LYN metastasis, *n* (%)	
No	126 (60.0)
Yes	84 (40.0)
Distance metastasis, *n* (%)	
No	210 (100.0)
Yes	0 (0.0)
TNM stage, *n* (%)	
I	36 (17.1)
II	87 (41.4)
III	87 (41.4)
CEA (ng/mL), median (IQR)	13.9 (2.9–45.7)
CA125 (U/mL), median (IQR)	35.1 (13.3–79.8)
Neoadjuvant chemotherapy, *n* (%)	81 (38.6)
Neoadjuvant chemotherapy regimen, *n* (%)	
NP	47 (58.0)
GP	14 (17.3)
DP	14 (17.3)
TP	6 (7.4)
Adjuvant chemotherapy, *n* (%)	162 (77.1)
Adjuvant chemotherapy regimen, *n* (%)	
NP	97 (59.9)
GP	24 (14.8)
DP	23 (14.2)
TP	18 (11.1)

Abbreviations: CA125; cancer antigen 125; CEA, carcinoembryonic antigen; DP, docetaxel plus cisplatin; ECOG PS, Eastern Cooperative Oncology Group Performance Status; GP, gemcitabine plus cisplatin; IQR, interquartile range; LYN, lymph node; NP, navelbine plus cisplatin; NSCLC, nonsmall cell lung cancer; SD, standard deviation; TNM, tumour‐nodes‐metastasis; TP, paclitaxel plus cisplatin.

### 3.2 Blood RGS1 expression in NSCLC patients (vs. HCs) and its correlation with clinical characteristics

**FIGURE 1:**
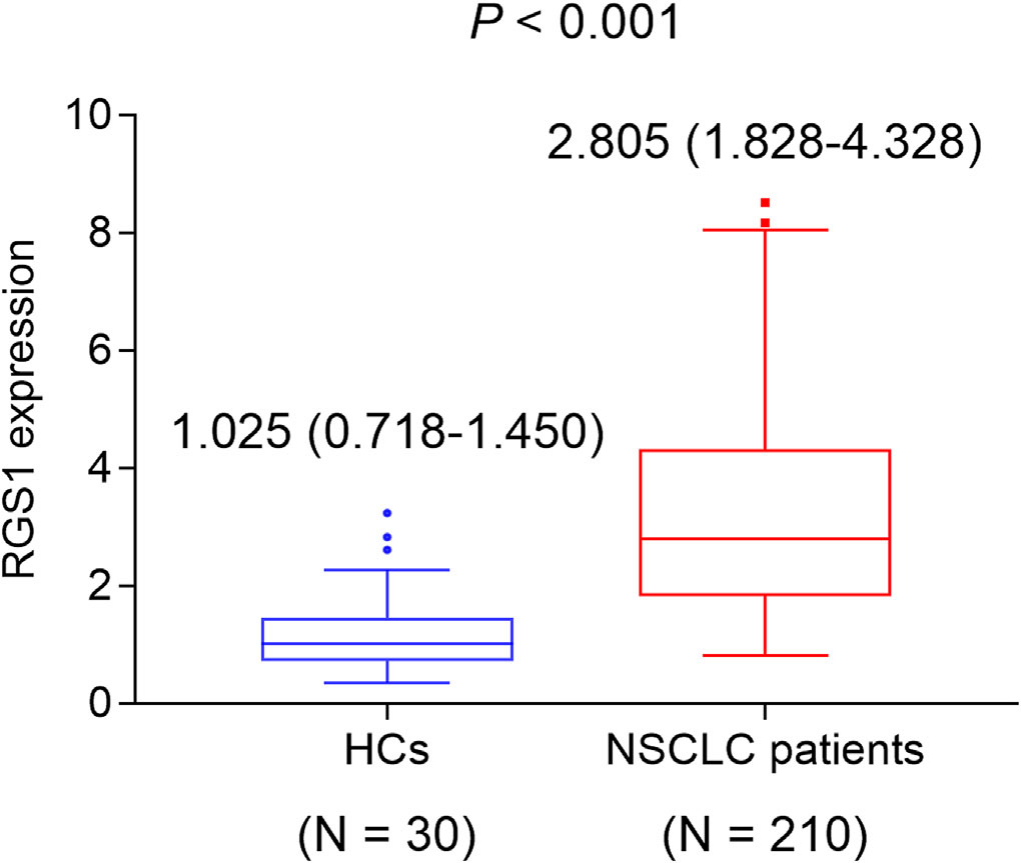
Blood RGS1 expression is elevated in NSCLC patients compared with HCs. NSCLC, nonsmall cell lung cancer; RGS1, regulator of G protein signalling 1.

Blood RGS1 expression was increased in NSCLC patients compared with HCs (median [interquartile range]: 2.805 [1.828–4.328] vs. 1.025 [0.718–1.450], *P* < 0.001, Figure 1).

With respect to the association of blood RGS1 with clinical characteristics of NSCLC patients, elevated blood RGS1 was related to lymph node (LYN) metastasis (*P* = 0.001), higher TNM stage (*P* = 0.004) and neoadjuvant chemotherapy application (*P* = 0.044), but it was not linked with age, gender, smoke, drink, hypertension, hyperlipidemia, diabetes, disease subtype, ECOG PS score, tumour differentiation, tumour size, carcinoembryonic antigen (CEA), cancer antigen 125 (CA125), neoadjuvant chemotherapy regimen, adjuvant chemotherapy application or adjuvant chemotherapy regimen (all *P* > 0.050) (Table 2).

**TABLE 2:** Correlation of RGS1 expression with clinical characteristics of NSCLC patients.

Item	RGS1 expression	*P*‐value
Age		0.323
≤ 60 years	2.610 (1.765–4.155)	
> 60 years	2.920 (1.945–4.510)	
Gender		0.786
Male	2.920 (1.825–4.220)	
Female	2.680 (1.815–4.635)	
Smoke		0.896
No	2.745 (1.833–4.298)	
Yes	2.895 (1.808–4.408)	
Drink		0.151
No	2.830 (1.950–4.590)	
Yes	2.780 (1.625–3.705)	
Hypertension		0.180
No	2.715 (1.868–4.073)	
Yes	3.480 (1.720–5.113)	
Hyperlipidemia		0.080
No	2.710 (1.760–4.185)	
Yes	3.295 (2.110–4.625)	
Diabetes		0.161
No	2.740 (1.805–4.265)	
Yes	3.470 (2.280–4.790)	
Subtype		0.595
Adenocarcinoma	2.945 (1.920–4.440)	
Squamous carcinoma	2.700 (1.820–4.135)	
Adenosquamous carcinoma	2.760 (1.120–4.650)	
ECOG PS score		0.312
0	2.745 (1.755–4.215)	
1	2.905 (2.095–4.590)	
Tumour differentiation		0.113
Well	2.700 (1.270–3.610)	
Moderate	2.980 (1.945–4.215)	
Poor	2.740 (1.890–5.270)	
Tumour size		0.252
≤ 5 cm	2.780 (1.740–4.100)	
> 5 cm	2.870 (1.950–4.500)	
LYN metastasis		0.001
No	2.600 (1.490–3.755)	
Yes	3.315 (2.350–4.720)	
Distant metastasis		‐
No	2.805 (1.828–4.328)	
Yes	‐	
TNM stage		0.004
I	2.925 (1.648–3.678)	
II	2.530 (1.490–4.130)	
III	3.380 (2.250–4.660)	
CEA		0.807
≤ 5 ng/mL	3.015 (1.703–4.155)	
> 5 ng/mL	2.770 (1.883–4.440)	
CA125		0.232
≤ 35 U/mL	2.720 (1.750–4.110)	
> 35 U/mL	3.070 (1.835–4.525)	
Neoadjuvant chemotherapy		0.044
No	2.700 (1.665–4.135)	
Yes	2.940 (2.160–4.595)	
Neoadjuvant chemotherapy regimen		0.516
NP	3.380 (2.550–4.630)	
GP	2.480 (1.658–4.593)	
DP	2.835 (1.460–4.578)	
TP	3.530 (2.558–5.225)	
Adjuvant chemotherapy		0.136
No	2.690 (1.548–3.678)	
Yes	2.845 (1.948–4.515)	
Adjuvant chemotherapy regimen		0.768
NP	2.960 (1.965–4.560)	
GP	2.620 (1.838–5.463)	
DP	2.720 (1.560–4.010)	
TP	2.795 (2.353–4.485)	

Abbreviations: CA199; cancer antigen 199; CEA, carcinoembryonic antigen; DP, docetaxel plus cisplatin; ECOG PS, Eastern Cooperative Oncology Group Performance Status; GP, gvemcitabine plus cisplatin; LYN, lymph node; NP, navelbine plus cisplatin; NSCLC, nonsmall cell lung cancer; RGS1, regulator of G protein signalling 1; TNM, tumour‐nodes‐metastasis; TP, paclitaxel plus cisplatin.

### 3.3 Correlation of blood RGS1 with survival in NSCLC patients

**FIGURE 2:**
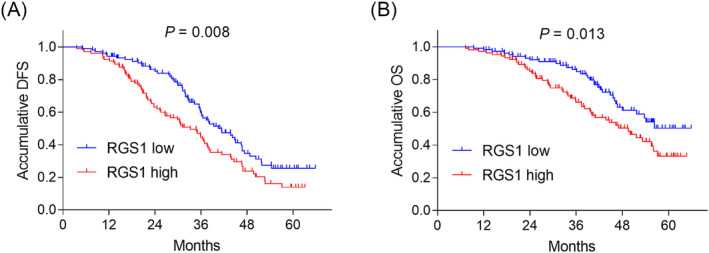
RGS1 high correlated with shortened DFS and OS in surgical NSCLC patients. Association of RGS1 with DFS (A) and OS (B) in NSCLC patients. DFS, disease‐free survival; NSCLC, nonsmall cell lung cancer; RGS1, regulator of G protein signalling 1.

Blood RGS1 high expression was associated with shortened accumulative DFS (*P* = 0.008, Figure 2A) and OS (*P* = 0.013, Figure 2B) in NSCLC patients. To further validate the prognostic value of blood RGS1, a multivariate Cox's regression analysis was performed, which showed that blood RGS1 high expression was independently correlated with shortened DFS (hazard ratio [HR] = 1.499, *P* = 0.023), whereas it was not related to OS (*P* > 0.050) (Table 3).

**TABLE 3:** Multivariate Cox's regression analysis for DFS and OS.

Item	*P*‐value	HR	95% CI	
			Lower limit	Upper limit
**DFS**				
RGS1 (high vs. low)	0.023	1.499	1.058	2.125
Poorer tumour differentiation	0.003	1.504	1.152	1.964
Higher TNM stage	0.001	2.120	1.352	3.325
Neoadjuvant chemotherapy (yes vs. no)	0.021	0.519	0.298	0.906
**OS**				
Age (>60 years vs. ≤60 years)	<0.001	2.607	1.645	3.646
Hypertension (yes vs. no)	0.007	2.032	1.214	3.402
Poorer tumour differentiation	<0.001	2.440	1.666	3.572
Higher TNM stage	<0.001	2.645	1.759	3.977

Abbreviations: CI, confidence interval; DFS, disease‐free survival; HR, hazard ratio; OS, overall survival; RGS1, regulator of G protein signalling 1; TNM, tumour‐nodes‐metastasis.

Besides, poorer tumour differentiation (HR = 1.504, *P* = 0.003) and higher TNM stage (HR = 2.120, *P* = 0.001) were independently related to shortened DFS; neoadjuvant chemotherapy (HR = 0.519, *P* = 0.021) was independently linked with prolonged DFS in NSCLC patients. Additionally, age >60 years (vs. ≤60 years) (HR = 2.607, *P* < 0.001), hypertension (HR = 2.032, *P* = 0.007), poorer tumour differentiation (HR = 2.440, *P* < 0.001) and higher TNM stage (HR = 2.645, *P* < 0.001) were independently associated with shortened OS (Table 3).

### 3.4 Best cutoff value of blood RGS1 for estimating recurrence and death events as well as subgroup analyses for suitable population

ROC curves showed that RGS1 potentially estimated recurrence events within 2 years (AUC = 0.671, 95% confidence interval [CI]: 0.585–0.758), 3 years (AUC = 0.628, 95% CI: 0.585–0.758), 4 years (AUC = 0.643, 95% CI: 0.569–0.717) and 5 years (AUC = 0.652, 95% CI: 0.578–0.727) as well as death events within 3 years (AUC = 0.681, 95% CI: 0.584–0.777), 4 years (AUC = 0.606, 95% CI: 0.525–0.688) and 5 years (AUC = 0.620, 95% CI: 0.542–0.698). But it could not estimate recurrence events within 1 year (AUC = 0.549, 95% CI: 0.394–0.703) as well as death events within 1 year (AUC = 0.513, 95% CI: 0.260–0.766) and 2 years (AUC = 0.611, 95% CI: 0.481–0.742). Meanwhile, the corresponding best cutoff value of RGS1 was listed in supporting information Table S1.

The best cutoff value of RGS1 for estimating 5‐year DFS and OS occurrence was 3.980 (sensitivity: 0.414, specificity: 0.870) and 3.685 (sensitivity: 0.463, specificity: 0.750). Subsequently, we utilised these two best cutoff values to perform further subgroup analyses. According to the subgroup analyses for DFS, patients with age >60 years, male patients, patients without hypertension, patients without hyperlipidemia, patients with moderate or poor tumour differentiation, patients with tumour size ≤ 5 cm, patients with LYN metastasis and patients with CEA > 5 ng/mL were potentially suitable populations to use RGS1 > 3.980 or RGS1 > 3.685 for estimating DFS (supporting information Table S2).

In terms of OS, patients with age >60 years, male patients, patients who drinked, patients without hypertension, patients with ECOG PS score 1, patients with moderate tumour differentiation, patients with LYN metastasis, patients with CEA > 5 ng/mL, patients with CA125 > 35 U/mL, patients with chemotherapy and patients with adjuvant chemotherapy were suitable populations to use RGS1 > 3.980 or RGS1 > 3.685 for estimating OS (supporting information Table S3).

### 3.5 Correlation of blood RGS1 with survival in NSCLC patients with/without neoadjuvant and adjuvant therapy

**FIGURE 3:**
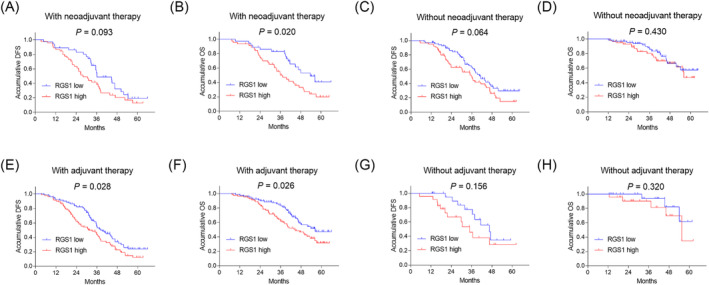
Prognostic value of RGS1 in surgical NSCLC patients with/without neoadjuvant or adjuvant therapy. Association of RGS1 with DFS (A) and OS (B) in patients with neoadjuvant therapy. Association of RGS1 with DFS (C) and OS (D) in patients without neoadjuvant therapy. Correlation of RGS1 with DFS (E) and OS (F) in patients with adjuvant therapy. Correlation of RGS1 with DFS (G) and OS (H) in patients without adjuvant therapy. DFS, disease‐free survival; NSCLC, nonsmall cell lung cancer; OS, overall survival; RGS1, regulator of G protein signalling 1.

Blood RGS1 was not linked with DFS (*P* = 0.093, Figure 3A), but blood RGS1 high expression was related to shortened OS (*P* = 0.020, Figure 3B) in patients with neoadjuvant therapy. Blood RGS1 was not associated with DFS (*P* = 0.064, Figure 3C) or OS (*P* = 0.430, Figure 3D) in patients without neoadjuvant therapy. Moreover, blood RGS1 high expression was correlated with shortened DFS (*P* = 0.028, Figure 3E) and OS (*P* = 0.026, Figure 3F) in patients with adjuvant therapy, while it was linked with neither DFS (*P* = 0.156, Figure 3G) nor OS (*P* = 0.320, Figure 3H) in patients without adjuvant therapy.

### 3.6 Public database‐analysis validation

With the help of the Gene Expression Profiling Interactive Analysis (GEPIA) database (http://gepia.cancer-pku.cn/detail.php?gene=RGS1), it was noticed that increased tumour RGS1 only exhibited a weak correlating trend (without statistical significance) with shortened DFS (*P* = 0.190, supporting information Figure S1A) and OS (*P* = 0.380, supporting information Figure S1B) in NSCLC patients; regrettably, blood RGS1 lacked relevant data. Then, Tumor Immune Estimation Resource (TIMER) database (https://cistrome.shinyapps.io/timer/) indicated that RGS1 was negatively correlated with cancer purity in both lung adenocarcinoma (LUAD) and lung squamous cell carcinoma (LUSC) (both *P* < 0.001, supporting information Figure S2). Furthermore, RGS1 was positively linked with the number of B cells, CD8^+^ T‐cells, CD4^+^ T‐cells, macrophages, neutrophils and dendritic cells in both LUAD and LUSC (all *P* < 0.001).

## 4 DISCUSSION

Previous studies have identified the increased blood RGS1 expression in many solid tumours, including hepatocellular carcinoma, renal cell carcinoma and ovarian cancer.^18^, ^19^ For instance, one study reveals that RGS1 is elevated in hepatocellular carcinoma at the initiation stage and tumourigenic stage compared with controls.^18^ Nonetheless, blood RGS1 expression in NSCLC patients still lacks determination. In this study, it was noticed that blood RGS1 expression was elevated in NSCLC patients compared with HCs, which could be explained by the following: RGS1 was considered an oncogene that is mainly expressed in B lymphocytes, T lymphocytes and other immune cells in the tumour microenvironment (TME); meanwhile, the immune cells were abundant and infiltrated more in NSCLC patients, leading to high expression of RGS1.^9^ Thus, blood RGS1 expression was heightened in NSCLC patients compared with HCs.

In addition to the aberrant expression, this study also found that increased blood RGS1 was linked with LYN metastasis, higher TNM stage and neoadjuvant chemotherapy administration in NSCLC patients. Probable explanations were those as follows: (1) Blood RGS1 partially flowed from the tumour tissue, thus increased blood RGS1 might reflect its high expression in tumour tissue, which indirectly promoted tumour malignant phenotypes and led to exacerbated tumour invasion.^9^ (2) Elevated blood RGS1 dysregulated the trafficking and functions of B and T lymphocytes in tumours and subsequently enabled tumour cells to escape immunosurveillance.^20^, ^21^ Due to the above two aspects, increased blood RGS1 was related to LYN metastasis and higher TNM stage in NSCLC patients. (3) As discussed above, elevated blood RGS1 was linked with a higher TNM stage; additionally, NSCLC patients with a higher TNM stage encountered more requests for neoadjuvant chemotherapy.^22^ As a result, elevated blood RGS1 was linked with neoadjuvant chemotherapy utilisation in NSCLC patients.

Concerning the prognostic value of RGS1, one previous study discloses that increased tumour RGS1 expression is related to poor DFS in ovarian cancer patients.^21^ Another study shows that the high expression of tumour RGS1 is correlated with shortened OS in gastric cancer patients.^16^ In the present study, blood RGS1 high expression was associated with shortened DFS and OS in surgical NSCLC patients; meanwhile, further multivariate Cox's regression analysis identified that blood RGS1 high expression independently revealed shortened DFS. Possible explanations were listed as follows: (1) As mentioned above, blood RGS1 high expression was related to elevated LYN metastasis, TNM stage, leading to worse prognosis in surgical NSCLC patients.^9^, ^18^, ^20^ (2) Aberrant expression of blood RGS1 enhanced TME immune tolerance and facilitated T‐cell exhaustion immune escape, which further caused tumour relapse of NSCLC.^12^ (3) Blood RGS1 dampened the antitumour response and promoted chemotherapy resistance.^23^ Combining the above aspects, blood RGS1 high expression was therefore related to shortened DFS and OS in surgical NSCLC patients. Furthermore, this study noticed that blood RGS1 high expression was linked with shortened DFS and OS in patients with neoadjuvant or adjuvant therapy, while their correlation was weak in patients without neoadjuvant or adjuvant therapy. This finding implied that the prognostic value of blood RGS1 might be attributed to its negative effect on chemotherapy sensitivity, which reduced the treatment efficacy of neoadjuvant or adjuvant therapy. Moreover, combining the findings of subgroup analyses, it could be summarised that patients with age >60 years, male patients, patients without hypertension, patients with moderate tumour differentiation, patients with LYN metastasis and patients with CEA > 5 ng/mL were suitable populations to use RGS1 > 3.980 or RGS1 > 3.685 for estimating DFS and OS.

Since the regulating role of RGS1 on the tumour immune microenvironment has been recognised recently, this study also investigated this issue through the TIMER database, which showed that RGS1 was negatively correlated with cancer purity.^9^, ^16^ The finding suggested that RGS1 was mainly expressed in immune cells rather than cancer cells, which was consistent with the previous study.^12^ Additionally, the TIMER database also suggested that RGS1 was positively associated with several tumour‐infiltrating immune cells, including B cells, CD8^+^ T‐cells, CD4^+^ T‐cells, macrophages, neutrophils and dendritic cells. A possible explanation was as follows: RGS1 was known to facilitate immune cell maturation and infiltration; thus, it was positively linked with the aforementioned tumour‐infiltrating immune cells in NSCLC patients.^24^, ^25^ Of note, the finding only implied the close correlation of RGS1 with immune infiltration, whereas the detailed internal relationship between RGS1 and each kind of immune cells in NSCLC patients should be verified in further studies.

Some limitations existed in the present study. Firstly, this was a single‐centre study, and the selective bias was therefore hard to avoid. Further multiple‐centre studies were warranted for verification. Secondly, this study collected blood RGS1 expression in NSCLC before treatment (neoadjuvant therapy if they had or surgery), while its variation during and after the treatment period was unknown, which deserved further investigation. Thirdly, further in vivo and in vitro studies were warranted for exploring the underlying mechanism of RGS1 in regulating tumourigenesis and tumour immunity of NSCLC. Fourthly, RGS1 expression in tumour specimen was not determined in this study, and it deserved further investigation of its consistency with blood RGS1 and its correlation with T‐cell exhaustion.

In summary, blood RGS1 is not only elevated in NSCLC patients, correlating with LYN metastasis and neoadjuvant chemotherapy application, but also possesses a good prognostic value that is meaningful for surgical NSCLC patient management.

## AUTHOR CONTRIBUTIONS

All authors contributed to the study conception and design. Material preparation, data collection and analysis were performed by L. W. and H. Z. Data interpretation and the first draft of the manuscript were written by L. W. and H. Z. All authors commented on previous versions of the manuscript. All authors read and approved the final manuscript.

## CONFLICT OF INTEREST STATEMENT

The authors have no relevant financial or nonfinancial interests to disclose.

## ETHICS STATEMENT

This study was reviewed by the Ethics Community of Baotou Cancer Hospital.

## PATIENT CONSENT STATEMENT

All participants signed informed consent.

## Supporting information


**Figure S1.** Survival analysis from GEPIA database. Association of tumour RGS1 with DFS (A) and OS (B) in NSCLC patients that analysed by GEPIA database.

**Figure S2.** Analysis from the TIMER database for evaluating the relationship between RGS1 and tumour‐infiltrating immune cells.

**Table S1.** Distinguish ability of RGS1 by ROC curve.

**Table S2.** Subgroup analysis for the correlation of RGS1 with DFS.

**Table S3.** Subgroup analysis for the correlation of RGS1 with OS.

## Data Availability

The datasets generated during and/or analysed during the current study are available from the corresponding author on reasonable request.

## References

[1] Rodak O , Peris‐Diaz MD , Olbromski M , Podhorska‐Okolow M , Dziegiel P . Current landscape of non‐small cell lung cancer: epidemiology, histological classification, targeted therapies, and immunotherapy. Cancers (Basel). 2021;13(18):13. doi:10.3390/cancers13184705 PMC8470525 34572931

[2] Sung H , Ferlay J , Siegel RL , et al. Global cancer statistics 2020: GLOBOCAN estimates of incidence and mortality worldwide for 36 cancers in 185 countries. CA Cancer J Clin. 2021;71(3):209‐249. doi:10.3322/caac.21660 33538338

[3] Chen P , Liu Y , Wen Y , Zhou C . Non‐small cell lung cancer in China. Cancer Commun (Lond). 2022;42(10):937‐970. doi:10.1002/cac2.12359 36075878 PMC9558689

[4] Alexander M , Kim SY , Cheng H . Update 2020: management of non‐small cell lung cancer. Lung. 2020;198(6):897‐907. doi:10.1007/s00408-020-00407-5 33175991 PMC7656891

[5] Ettinger DS , Wood DE , Aisner DL , et al. Non‐small cell lung cancer, version 3.2022, NCCN clinical practice guidelines in oncology. J Natl Compr Canc Netw. 2022;20(5):497‐530. doi:10.6004/jnccn.2022.0025 35545176

[6] Duma N , Santana‐Davila R , Molina JR . Non‐small cell lung cancer: epidemiology, screening, diagnosis, and treatment. Mayo Clin Proc. 2019;94(8):1623‐1640. doi:10.1016/j.mayocp.2019.01.013 31378236

[7] Goldstraw P , Chansky K , Crowley J , et al. The IASLC lung cancer staging project: proposals for revision of the TNM stage groupings in the forthcoming (eighth) edition of the TNM classification for lung cancer. J Thorac Oncol. 2016;11(1):39‐51. doi:10.1016/j.jtho.2015.09.009 26762738

[8] Feng Z , Zhou J , Liu Y , et al. Epithelium‐ and endothelium‐derived exosomes regulate the alveolar macrophages by targeting RGS1 mediated calcium signaling‐dependent immune response. Cell Death Differ. 2021;28(7):2238‐2256. doi:10.1038/s41418-021-00750-x 33753901 PMC8257848

[9] Zhang S , Wang H , Liu J , Tao T , Zeng Z , Wang M . RGS1 and related genes as potential targets for immunotherapy in cervical cancer: computational biology and experimental validation. J Transl Med. 2022;20(1):334. doi:10.1186/s12967-022-03526-0 35879796 PMC9310486

[10] Zhang L , Yao M , Ma W , Jiang Y , Wang W . MicroRNA‐376b‐3p targets RGS1 mRNA to inhibit proliferation, metastasis, and apoptosis in osteosarcoma. Ann Transl Med. 2021;9(22):1652. doi:10.21037/atm-21-4949 34988161 PMC8667113

[11] Bai Y , Hu M , Chen Z , Wei J , Du H . Single‐cell transcriptome analysis reveals RGS1 as a new marker and promoting factor for T‐cell exhaustion in multiple cancers. Front Immunol. 2021;12:767070. doi:10.3389/fimmu.2021.767070 34956194 PMC8692249

[12] Huang D , Chen X , Zeng X , et al. Targeting regulator of G protein signaling 1 in tumor‐specific T cells enhances their trafficking to breast cancer. Nat Immunol. 2021;22(7):865‐879. doi:10.1038/s41590-021-00939-9 34140678

[13] Carreras J , Kikuti YY , Bea S , et al. Clinicopathological characteristics and genomic profile of primary sinonasal tract diffuse large B cell lymphoma (DLBCL) reveals gain at 1q31 and RGS1 encoding protein; high RGS1 immunohistochemical expression associates with poor overall survival in DLBCL not otherwise specified (NOS). Histopathology. 2017;70(4):595‐621. doi:10.1111/his.13106 27775850

[14] Dai J , Gu J , Lu C , et al. Genetic variations in the regulator of G‐protein signaling genes are associated with survival in late‐stage non‐small cell lung cancer. PLoS ONE. 2011;6(6):e21120. doi:10.1371/journal.pone.0021120 21698121 PMC3117866

[15] Livak KJ , Schmittgen TD . Analysis of relative gene expression data using real‐time quantitative PCR and the 2(‐Delta Delta C[T]) method. Methods. 2001;25(4):402‐408. doi:10.1006/meth.2001.1262 11846609

[16] Li S , Yang H , Li S , Zhao Z , Wang D , Fu W . High expression of regulator of G‐protein signalling 1 is associated with the poor differentiation and prognosis of gastric cancer. Oncol Lett. 2021;21(4):322. doi:10.3892/ol.2021.12584 33692854 PMC7933750

[17] Garson JA , Usher L , Al‐Chalabi A , Huggett J , Day EF , McCormick AL . Quantitative analysis of human endogenous retrovirus‐K transcripts in postmortem premotor cortex fails to confirm elevated expression of HERV‐K RNA in amyotrophic lateral sclerosis. Acta Neuropathol Commun. 2019;7(1):45. doi:10.1186/s40478-019-0698-2 30885274 PMC6421708

[18] Ranjpour M , Wajid S , Jain SK . Elevated expression of sepiapterin reductase, regulator of G protein signaling 1, hypothetical protein CXorf58 homolog, and zinc finger and BTB domain‐containing protein 21 isoform X2 is associated with progression of hepatocellular carcinoma. Protoplasma. 2021;258(5):1133‐1143. doi:10.1007/s00709-021-01632-2 33683453

[19] Sethakorn N , Dulin NO . RGS expression in cancer: oncomining the cancer microarray data. J Recept Signal Transduct Res. 2013;33(3):166‐171. doi:10.3109/10799893.2013.773450 23464602

[20] Sun MY , Wang Y , Zhu J , et al. Critical role for non‐GAP function of Galphas in RGS1‐mediated promotion of melanoma progression through AKT and ERK phosphorylation. Oncol Rep. 2018;39(6):2673‐2680. doi:10.3892/or.2018.6341 29620236

[21] Hu Y , Zheng M , Wang S , et al. Identification of a five‐gene signature of the RGS gene family with prognostic value in ovarian cancer. Genomics. 2021;113(4):2134‐2144. doi:10.1016/j.ygeno.2021.04.012 33845140

[22] Ettinger DS , Wood DE , Aisner DL , et al. NCCN guidelines insights: non‐small cell lung cancer, version 2.2021. J Natl Compr Canc Netw. 2021;19(3):254‐266. doi:10.6004/jnccn.2021.0013 33668021

[23] Zhou K , Hu N , Hong Y , et al. An immune‐related prognostic signature predicts overall survival in stomach adenocarcinomas. Front Genet. 2022;13:903393. doi:10.3389/fgene.2022.903393 35677557 PMC9168657

[24] Pak HK , Gil M , Lee Y , et al. Regulator of G protein signaling 1 suppresses CXCL12‐mediated migration and AKT activation in RPMI 8226 human plasmacytoma cells and plasmablasts. PLoS ONE. 2015;10(4):e0124793. doi:10.1371/journal.pone.0124793 25897806 PMC4405207

[25] Zhao H , Dang R , Zhu Y , et al. Hub genes associated with immune cell infiltration in breast cancer, identified through bioinformatic analyses of multiple datasets. Cancer Biol Med. 2022;19(9):1352‐1374. doi:10.20892/j.issn.2095-3941.2021.0586 35819135 PMC9500228

