# Supplementary figures and images for: Blood regulator of G protein signalling 1 as a potential prognostic biomarker in surgical nonsmall cell lung cancer patients: Correlation with clinical features and survival
Source: Clin Respir J. 2023 Dec 11;18(1):e13712. doi: 10.1111/crj.13712 (PMC10807578; doi:10.1111/crj.13712)

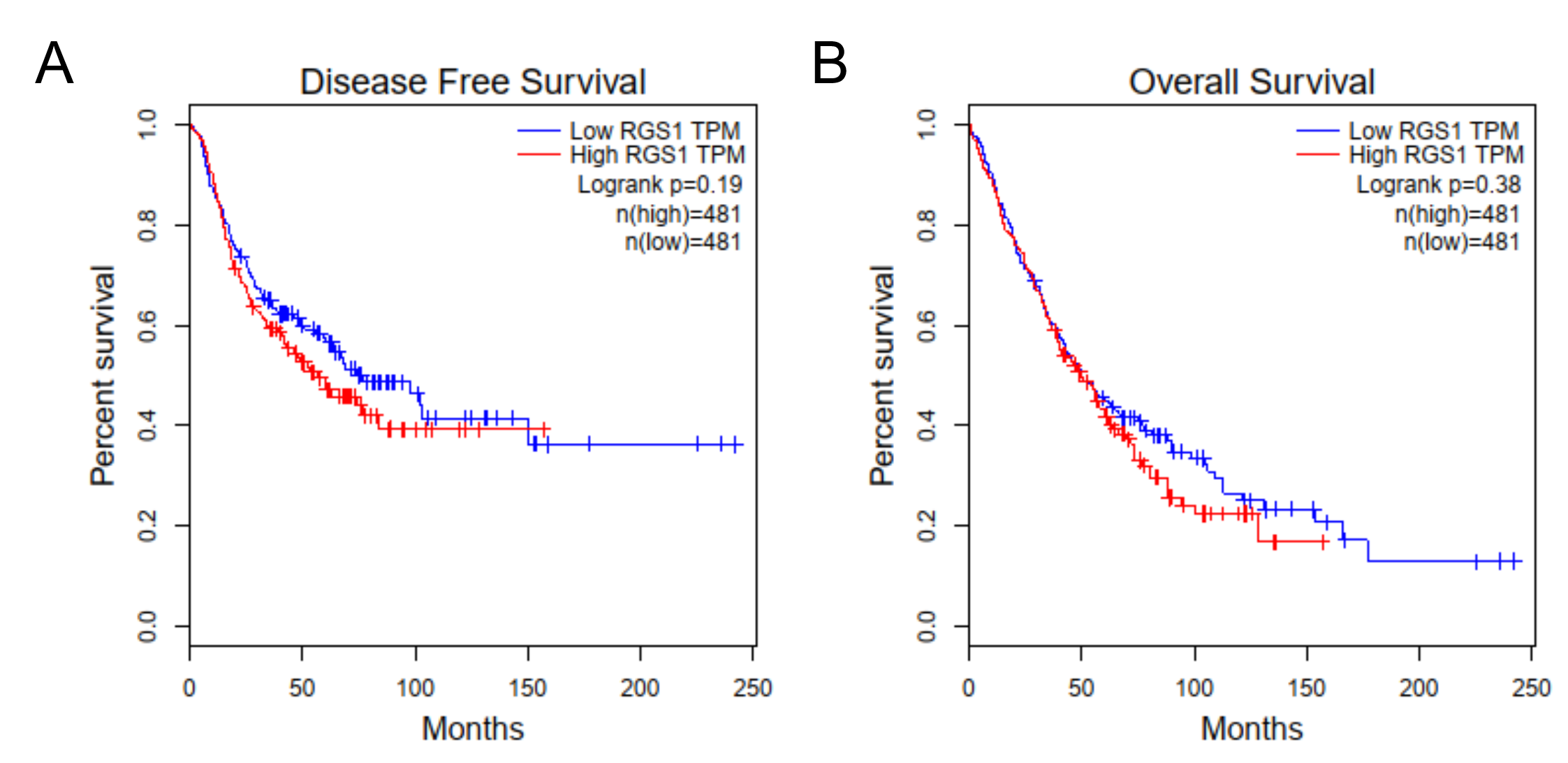

Supplement: Supplementary file 1 — Figure S1. Survival analysis from GEPIA database. Association of tumour RGS1 with DFS (A) and OS (B) in NSCLC patients that analysed by GEPIA database. [file CRJ-18-e13712-s005.tif]

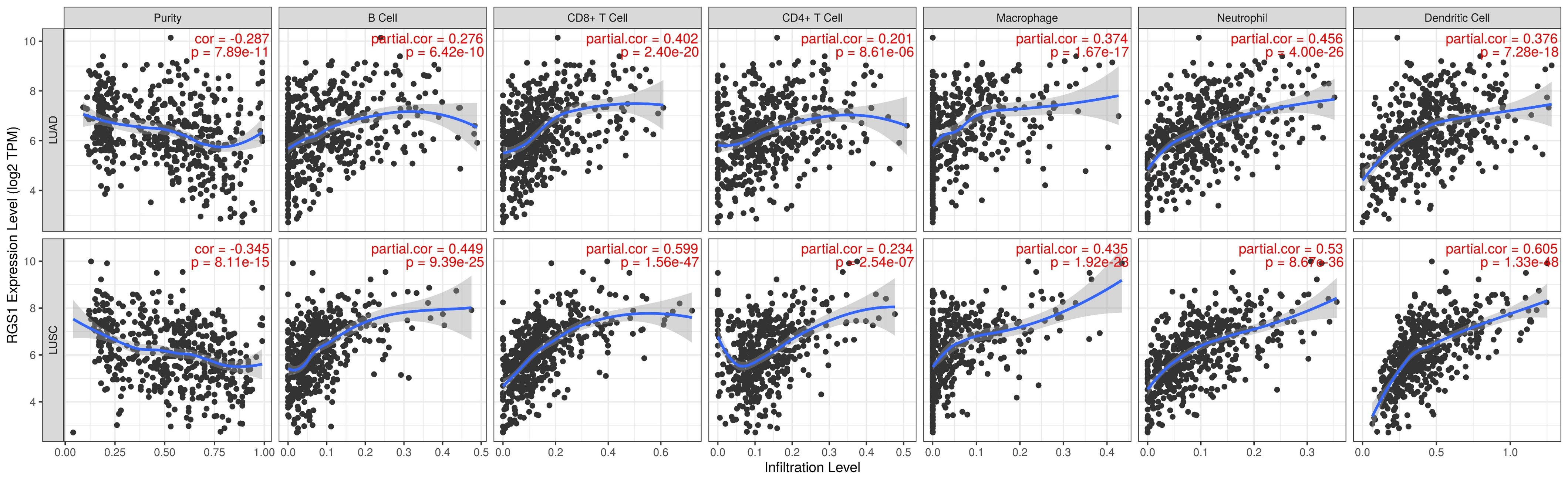

Supplement: Supplementary file 2 — Figure S2. Analysis from the TIMER database for evaluating the relationship between RGS1 and tumour‐infiltrating immune cells. [file CRJ-18-e13712-s003.tif]
