# Supplementary material for: Blood regulator of G protein signalling 1 as a potential prognostic biomarker in surgical nonsmall cell lung cancer patients: Correlation with clinical features and survival
Source: Clin Respir J. 2023 Dec 11;18(1):e13712. doi: 10.1111/crj.13712 (PMC10807578; doi:10.1111/crj.13712)
Supplement: Supplementary file 3 — Table S1. Distinguish ability of RGS1 by ROC curve. [file CRJ-18-e13712-s002.docx]

**Supplementary Table 1.** Distinguish ability of RGS1 by ROC curve.

| Items | AUC (95% CI) | Best cut-off value | Sensitivity | Specificity |
| --- | --- | --- | --- | --- |
| **Recurrence** **(vs. none recurrence)** | | | | |
| 1-year | 0.549 (0.394-0.703) | 2.035 | 0.857 | 0.316 |
| 2-year | 0.671 (0.585-0.758) | 3.015 | 0.692 | 0.614 |
| 3-year | 0.628 (0.551-0.705) | 3.980 | 0.433 | 0.783 |
| 4-year | 0.643 (0.569-0.717) | 4.625 | 0.317 | 0.943 |
| 5-year | 0.652 (0.578-0.727) | 3.980 | 0.414 | 0.870 |
| **Death (vs. survivor)** | | | | |
| 1-year | 0.513 (0.260-0.766) | 2.425 | 0.800 | 0.376 |
| 2-year | 0.611 (0.481-0.742) | 3.980 | 0.565 | 0.722 |
| 3-year | 0.681 (0.584-0.777) | 3.980 | 0.591 | 0.765 |
| 4-year | 0.606 (0.525-0.688) | 3.525 | 0.493 | 0.691 |
| 5-year | 0.620 (0.542-0.698) | 3.685 | 0.463 | 0.750 |

ROC, receiver-operating characteristic; AUC, area under the curve; CI, confidence interval.
