# Supplementary material for: Blood regulator of G protein signalling 1 as a potential prognostic biomarker in surgical nonsmall cell lung cancer patients: Correlation with clinical features and survival
Source: Clin Respir J. 2023 Dec 11;18(1):e13712. doi: 10.1111/crj.13712 (PMC10807578; doi:10.1111/crj.13712)
Supplement: Supplementary file 4 — Table S2. Subgroup analysis for the correlation of RGS1 with DFS. [file CRJ-18-e13712-s004.docx]

**Supplementary Table 2.** Subgroup analysis for the correlation of RGS1 with DFS.

| Items | RGS1 >3.980 vs. ≤3.980 | | | | RGS1 >3.685 vs. ≤3.685 | | | |
| --- | --- | --- | --- | --- | --- | --- | --- | --- |
|  | *P* value | HR | 95% CI | | *P* value | HR | 95% CI | |
|  |  |  | Lower limit | Upper limit |  |  | Lower limit | Upper limit |
| Age |  |  |  |  |  |  |  |  |
| ≤ 60 years | 0.088 | 1.625 | 0.931 | 2.836 | 0.276 | 1.362 | 0.781 | 2.376 |
| > 60 years | <0.001 | 2.378 | 1.524 | 3.711 | <0.001 | 2.229 | 1.432 | 3.469 |
| Gender |  |  |  |  |  |  |  |  |
| Male | <0.001 | 2.197 | 1.496 | 3.227 | <0.001 | 2.065 | 1.409 | 3.026 |
| Female | 0.275 | 1.572 | 0.697 | 3.544 | 0.623 | 1.227 | 0.543 | 2.774 |
| Smoke |  |  |  |  |  |  |  |  |
| No | 0.001 | 2.108 | 1.331 | 3.337 | 0.003 | 1.985 | 1.259 | 3.130 |
| Yes | 0.016 | 1.921 | 1.129 | 3.267 | 0.079 | 1.604 | 0.947 | 2.717 |
| Drink |  |  |  |  |  |  |  |  |
| No | 0.005 | 1.822 | 1.198 | 2.773 | 0.011 | 1.717 | 1.129 | 2.611 |
| Yes | <0.001 | 4.332 | 2.143 | 8.755 | 0.003 | 2.670 | 1.412 | 5.049 |
| Hypertension |  |  |  |  |  |  |  |  |
| No | <0.001 | 2.507 | 1.644 | 3.823 | <0.001 | 2.319 | 1.525 | 3.528 |
| Yes | 0.479 | 1.255 | 0.669 | 2.354 | 0.813 | 1.079 | 0.574 | 2.029 |
| Hyperlipidemia |  |  |  |  |  |  |  |  |
| No | <0.001 | 2.230 | 1.450 | 3.429 | <0.001 | 2.321 | 1.512 | 3.564 |
| Yes | 0.073 | 1.708 | 0.950 | 3.071 | 0.577 | 1.181 | 0.658 | 2.121 |
| Diabetes |  |  |  |  |  |  |  |  |
| No | 0.003 | 1.809 | 1.228 | 2.666 | 0.028 | 1.547 | 1.049 | 2.281 |
| Yes | 0.005 | 3.270 | 1.443 | 7.409 | 0.002 | 3.811 | 1.637 | 8.870 |
| Subtype |  |  |  |  |  |  |  |  |
| Adenocarcinoma | 0.012 | 1.868 | 1.148 | 3.041 | 0.102 | 1.501 | 0.923 | 2.441 |
| Squamous carcinoma | 0.003 | 2.212 | 1.312 | 3.732 | 0.002 | 2.272 | 1.351 | 3.822 |
| Adenosquamous carcinoma | 0.961 | 1.044 | 0.184 | 5.920 | 0.961 | 1.044 | 0.184 | 5.920 |
| ECOG PS score |  |  |  |  |  |  |  |  |
| 0 | 0.007 | 1.777 | 1.170 | 2.700 | 0.033 | 1.568 | 1.036 | 2.374 |
| 1 | 0.005 | 2.587 | 1.334 | 5.017 | 0.005 | 2.587 | 1.334 | 5.017 |
| Tumor differentiation |  |  |  |  |  |  |  |  |
| Well | 0.269 | 0.314 | 0.040 | 2.447 | 0.146 | 0.218 | 0.028 | 1.698 |
| Moderate | <0.001 | 2.492 | 1.562 | 3.976 | <0.001 | 2.327 | 1.463 | 3.703 |
| Poor | 0.018 | 2.008 | 1.129 | 3.569 | 0.018 | 2.008 | 1.129 | 3.569 |
| Tumor size |  |  |  |  |  |  |  |  |
| ≤ 5 cm | <0.001 | 2.850 | 1.657 | 4.903 | <0.001 | 3.037 | 1.769 | 5.214 |
| > 5 cm | 0.064 | 1.530 | 0.975 | 2.401 | 0.380 | 1.223 | 0.780 | 1.916 |
| LYN metastasis |  |  |  |  |  |  |  |  |
| No | 0.104 | 1.551 | 0.914 | 2.633 | 0.283 | 1.332 | 0.789 | 2.248 |
| Yes | 0.002 | 2.140 | 1.321 | 3.467 | 0.002 | 2.122 | 1.309 | 3.441 |
| Distant metastasis |  |  |  |  |  |  |  |  |
| No | <0.001 | 2.036 | 1.440 | 2.877 | 0.001 | 1.836 | 1.301 | 2.590 |
| Yes | - | - | - | - | - | - | - | - |
| TNM stage |  |  |  |  |  |  |  |  |
| I | 0.020 | 4.976 | 1.289 | 19.211 | 0.003 | 7.705 | 1.979 | 30.001 |
| II | 0.507 | 1.214 | 0.685 | 2.153 | 0.445 | 1.245 | 0.709 | 2.185 |
| III | 0.002 | 2.132 | 1.311 | 3.468 | 0.060 | 1.594 | 0.981 | 2.590 |
| CEA |  |  |  |  |  |  |  |  |
| ≤ 5 ng/mL | 0.124 | 1.598 | 0.880 | 2.903 | 0.354 | 1.318 | 0.735 | 2.365 |
| > 5 ng/mL | <0.001 | 2.206 | 1.438 | 3.382 | <0.001 | 2.206 | 1.438 | 3.382 |
| CA125 |  |  |  |  |  |  |  |  |
| ≤ 35 U/mL | 0.012 | 1.957 | 1.161 | 3.297 | 0.110 | 1.526 | 0.909 | 2.561 |
| > 35 U/mL | 0.005 | 1.951 | 1.221 | 3.116 | 0.003 | 2.035 | 1.274 | 3.249 |
| Neoadjuvant chemotherapy |  |  |  |  |  |  |  |  |
| No | 0.031 | 1.692 | 1.050 | 2.725 | 0.015 | 1.792 | 1.121 | 2.865 |
| Yes | 0.001 | 2.417 | 1.439 | 4.061 | 0.027 | 1.795 | 1.070 | 3.010 |
| Adjuvant chemotherapy |  |  |  |  |  |  |  |  |
| No | 0.006 | 3.508 | 1.439 | 8.553 | 0.002 | 4.145 | 1.722 | 9.975 |
| Yes | 0.002 | 1.831 | 1.257 | 2.669 | 0.018 | 1.574 | 1.081 | 2.290 |

DFS, disease-free survival; RGS1, regulator of G protein signaling 1; HR, hazard ratio; CI, confidence interval; ECOG PS, Eastern Cooperative Oncology Group Performance Status; LYN, lymph node; TNM, tumor nodes metastasis; CEA, carcinoembryonic antigen; CA199; cancer antigen 199; NP, navelbine plus cisplatin; TP, paclitaxel plus cisplatin; GP, gemcitabine plus cisplatin; DP, docetaxel plus cisplatin.
