# Supplementary material for: Blood regulator of G protein signalling 1 as a potential prognostic biomarker in surgical nonsmall cell lung cancer patients: Correlation with clinical features and survival
Source: Clin Respir J. 2023 Dec 11;18(1):e13712. doi: 10.1111/crj.13712 (PMC10807578; doi:10.1111/crj.13712)
Supplement: Supplementary file 5 — Table S3. Subgroup analysis for the correlation of RGS1 with OS. [file CRJ-18-e13712-s001.docx]

**Supplementary Table 3.** Subgroup analysis for the correlation of RGS1 with OS.

| Items | RGS1 >3.980 vs. ≤3.980 | | | | RGS1 >3.685 vs. ≤3.685 | | | |
| --- | --- | --- | --- | --- | --- | --- | --- | --- |
|  | *P* value | HR | 95% CI | | *P* value | HR | 95% CI | |
|  |  |  | Lower limit | Upper limit |  |  | Lower limit | Upper limit |
| Age |  |  |  |  |  |  |  |  |
| ≤ 60 years | 0.883 | 0.935 | 0.386 | 2.268 | 0.620 | 0.799 | 0.330 | 1.937 |
| > 60 years | 0.001 | 2.489 | 1.485 | 4.172 | 0.001 | 2.477 | 1.477 | 4.155 |
| Gender |  |  |  |  |  |  |  |  |
| Male | 0.010 | 1.876 | 1.165 | 3.022 | 0.008 | 1.894 | 1.179 | 3.042 |
| Female | 0.187 | 2.107 | 0.697 | 6.368 | 0.361 | 1.680 | 0.552 | 5.114 |
| Smoke |  |  |  |  |  |  |  |  |
| No | 0.046 | 1.816 | 1.010 | 3.264 | 0.039 | 1.841 | 1.032 | 3.284 |
| Yes | 0.030 | 2.092 | 1.075 | 4.071 | 0.065 | 1.874 | 0.963 | 3.647 |
| Drink |  |  |  |  |  |  |  |  |
| No | 0.050 | 1.735 | 1.000 | 3.010 | 0.059 | 1.699 | 0.979 | 2.949 |
| Yes | 0.003 | 3.222 | 1.504 | 6.901 | 0.009 | 2.661 | 1.273 | 5.564 |
| Hypertension |  |  |  |  |  |  |  |  |
| No | 0.010 | 2.000 | 1.181 | 3.388 | 0.013 | 1.944 | 1.152 | 3.280 |
| Yes | 0.132 | 1.865 | 0.829 | 4.198 | 0.166 | 1.787 | 0.786 | 4.064 |
| Hyperlipidemia |  |  |  |  |  |  |  |  |
| No | 0.045 | 1.751 | 1.013 | 3.027 | 0.027 | 1.849 | 1.073 | 3.186 |
| Yes | 0.033 | 2.211 | 1.065 | 4.593 | 0.119 | 1.789 | 0.861 | 3.716 |
| Diabetes |  |  |  |  |  |  |  |  |
| No | 0.036 | 1.688 | 1.035 | 2.752 | 0.110 | 1.491 | 0.914 | 2.433 |
| Yes | 0.045 | 2.897 | 1.024 | 8.199 | 0.017 | 4.061 | 1.289 | 12.798 |
| Subtype |  |  |  |  |  |  |  |  |
| Adenocarcinoma | 0.044 | 1.893 | 1.016 | 3.525 | 0.100 | 1.684 | 0.905 | 3.133 |
| Squamous carcinoma | 0.031 | 2.063 | 1.067 | 3.990 | 0.021 | 2.160 | 1.123 | 4.155 |
| Adenosquamous carcinoma | 0.897 | 1.144 | 0.150 | 8.697 | 0.897 | 1.144 | 0.150 | 8.697 |
| ECOG PS score |  |  |  |  |  |  |  |  |
| 0 | 0.052 | 1.740 | 0.996 | 3.040 | 0.064 | 1.685 | 0.971 | 2.924 |
| 1 | 0.038 | 2.163 | 1.043 | 4.485 | 0.038 | 2.163 | 1.043 | 4.485 |
| Tumor differentiation |  |  |  |  |  |  |  |  |
| Well | 0.671 | 0.624 | 0.071 | 5.484 | 0.479 | 0.455 | 0.051 | 4.029 |
| Moderate | 0.005 | 2.417 | 1.299 | 4.499 | 0.004 | 2.525 | 1.353 | 4.712 |
| Poor | 0.326 | 1.395 | 0.718 | 2.714 | 0.326 | 1.395 | 0.718 | 2.714 |
| Tumor size |  |  |  |  |  |  |  |  |
| ≤ 5 cm | 0.061 | 2.260 | 0.962 | 5.309 | 0.028 | 2.573 | 1.108 | 5.974 |
| > 5 cm | 0.072 | 1.598 | 0.959 | 2.663 | 0.225 | 1.371 | 0.823 | 2.282 |
| LYN metastasis |  |  |  |  |  |  |  |  |
| No | 0.326 | 0.639 | 0.262 | 1.561 | 0.283 | 0.630 | 0.271 | 1.466 |
| Yes | 0.001 | 2.666 | 1.508 | 4.713 | 0.001 | 2.754 | 1.549 | 4.896 |
| Distant metastasis |  |  |  |  |  |  |  |  |
| No | 0.004 | 1.898 | 1.226 | 2.938 | 0.007 | 1.826 | 1.182 | 2.821 |
| Yes | - | - | - | - | - | - | - | - |
| TNM stage |  |  |  |  |  |  |  |  |
| I | 0.738 | 0.041 | <0.001 | 5469017.864 | 0.281 | 4.619 | 0.287 | 74.375 |
| II | 0.691 | 0.829 | 0.329 | 2.089 | 0.956 | 0.976 | 0.406 | 2.347 |
| III | 0.008 | 2.083 | 1.215 | 3.572 | 0.064 | 1.666 | 0.971 | 2.857 |
| CEA |  |  |  |  |  |  |  |  |
| ≤ 5 ng/mL | 0.165 | 1.909 | 0.766 | 4.757 | 0.116 | 2.063 | 0.836 | 5.088 |
| > 5 ng/mL | 0.015 | 1.857 | 1.128 | 3.056 | 0.015 | 1.857 | 1.128 | 3.056 |
| CA125 |  |  |  |  |  |  |  |  |
| ≤ 35 U/mL | 0.130 | 1.763 | 0.846 | 3.673 | 0.223 | 1.565 | 0.761 | 3.215 |
| > 35 U/mL | 0.027 | 1.869 | 1.073 | 3.257 | 0.017 | 1.964 | 1.127 | 3.424 |
| Neoadjuvant chemotherapy |  |  |  |  |  |  |  |  |
| No | 0.849 | 1.074 | 0.514 | 2.245 | 0.446 | 1.313 | 0.652 | 2.644 |
| Yes | 0.002 | 2.465 | 1.384 | 4.390 | 0.023 | 1.951 | 1.096 | 3.475 |
| Adjuvant chemotherapy |  |  |  |  |  |  |  |  |
| No | 0.708 | 1.360 | 0.273 | 6.775 | 0.275 | 2.223 | 0.529 | 9.341 |
| Yes | 0.009 | 1.838 | 1.162 | 2.907 | 0.025 | 1.686 | 1.067 | 2.665 |

OS, overall survival; RGS1, regulator of G protein signaling 1; HR, hazard ratio; CI, confidence interval; ECOG PS, Eastern Cooperative Oncology Group Performance Status; LYN, lymph node; TNM, tumor nodes metastasis; CEA, carcinoembryonic antigen; CA199; cancer antigen 199; NP, navelbine plus cisplatin; TP, paclitaxel plus cisplatin; GP, gemcitabine plus cisplatin; DP, docetaxel plus cisplatin.
